# The ulcer that would not heal: a rare cutaneous clue to miliary tuberculosis

**DOI:** 10.1093/skinhd/vzag011

**Published:** 2026-04-02

**Authors:** Elisha Shrestha, Ashish Tamang, Namrata Pradhan, Sanjan Kumar Sah, Binay Yadav, Anupa Sharma

**Affiliations:** Department of Dermatology, Dhulikhel Hospital, Kathmandu University Hospital, Kathmandu, Nepal; Department of Dermatology, Dhulikhel Hospital, Kathmandu University Hospital, Kathmandu, Nepal; Department of Dermatology, Cutis Care, Kathmandu, Nepal; Department of Dermatology, Dhulikhel Hospital, Kathmandu University Hospital, Kathmandu, Nepal; Department of Dermatology, Dhulikhel Hospital, Kathmandu University Hospital, Kathmandu, Nepal; Department of Dermatology, Dhulikhel Hospital, Kathmandu University Hospital, Kathmandu, Nepal

## Abstract

Cutaneous tuberculosis (TB) is a rare manifestation of extrapulmonary TB, accounting for only 1–1.5% of cases. Among its various forms, cutaneous miliary TB is exceptionally uncommon and often presents with nonspecific clinical features. This makes early diagnosis challenging, especially in individuals who are immunocompromised. We report the case of a 67-year-old woman who presented with a 6-month history of multiple painful, nonhealing ulcers over the mons pubis, perivulvar area and left upper arm. Despite multiple courses of antibiotics, there was no improvement. Clinical examination revealed multiple well-defined ulcers with yellowish discharge. The patient had no history of bacille Calmette–Guérin vaccination. Tissue GeneXpert analysis from the ulcer confirmed *Mycobacterium tuberculosis*, and chest radiography revealed bilateral pulmonary nodules consistent with miliary TB. Imaging of the spine also showed features of spinal TB. The patient was diagnosed with miliary TB and was initiated on standard antitubercular therapy. Significant clinical improvement was noted within 1 month of treatment. This case highlights the importance of considering cutaneous TB, including its rare miliary form, in the differential diagnosis of chronic nonhealing ulcers, particularly in endemic areas or in patients with systemic symptoms. Early diagnosis and appropriate therapy are crucial for favourable outcomes.

## Teaser

This case report describes a 67-year-old woman who presented with multiple nonhealing ulcers over the mons pubis, perivulvar area and left upper arm, unresponsive to antibiotics. Investigations confirmed cutaneous miliary tuberculosis with pulmonary and spinal involvement (Pott spine). The patient showed significant clinical improvement after 1 month of standard antitubercular therapy. This case underscores the importance of considering cutaneous tuberculosis, particularly its rare miliary form, in the differential diagnosis of chronic ulcers, especially in endemic regions and in patients who are immunocompromised.

What is already known about this topic?

- Cutaneous tuberculosis (TB) is a rare form of extrapulmonary TB, accounting for only 1–1.5% of all TB cases.
- Among its various clinical manifestations, miliary cutaneous TB is exceptionally uncommon and typically occurs secondary to haematogenous dissemination of *Mycobacterium tuberculosis* from a primary internal focus; it often presents with nonspecific clinical features such as multiple papules, pustules or ulcers, which can mimic other infectious or inflammatory dermatoses.
- The diagnosis is particularly challenging due to its rarity, atypical presentation and the need for high clinical suspicion, especially in individuals who are immunocompromised; histopathology, acid-fast bacilli staining, culture and molecular methods like polymerase chain reaction are often required for confirmation.

What does this study add?

- This report describes a rare instance of cutaneous miliary TB in an older woman who developed several nonhealing ulcers over her left upper arm, vulvar region and perivulvar region; the unusual distribution and chronicity of the lesions mimicked other infectious or inflammatory dermatoses, contributing to diagnostic delay.
- This report emphasizes the need for high clinical suspicion for cutaneous TB, even in patients without overt systemic symptoms or immunosuppression; it also reinforces the role of histopathology, acid-fast bacilli staining and molecular diagnostics in confirming rare forms of TB.
- The case highlights the diagnostic challenges in endemic yet resource-limited settings and encourages clinicians to consider cutaneous TB in the differential diagnosis of chronic ulcers with atypical distribution.

Tuberculosis (TB) remains one of the leading causes of mortality and morbidity worldwide, with an estimated 10.8 million new cases and 1.25 million deaths reported in 2023.^1^ Pulmonary TB is the most common form; however, extrapulmonary TB accounts for 15–20% of cases, and is particularly common among individuals who are immunocompromised.^2^ With the increasing prevalence of diabetes, malnutrition and other causes of immunosuppression, TB now presents with more atypical and unusual forms. Cutaneous TB is a rare form of extrapulmonary TB, representing only 1.5–3% of cases worldwide. *Mycobacterium tuberculosis* is the most common cause of cutaneous TB; however, *Mycobacterium bovis* and bacille Calmette–Guérin (BCG) vaccine are rare causes of the disease.^3–5^ TB may occur through direct inoculation, contiguous extension from underlying focus or haematogenous spread of *M. tuberculosis* during systemic infection.^6^ It often mimics other bacterial, fungal or neoplastic conditions because of its uncommon and varied morphology. This makes the diagnosis challenging and requires a high index of suspicion.^6^ Early recognition is crucial, as cutaneous involvement may signal disseminated or miliary TB, a potentially life-threatening condition if left untreated.^7^ Delay in diagnosis can lead to severe complications, including skeletal or visceral involvement.

Here, we present the case of a 67-year-old woman with nonhealing cutaneous ulcers secondary to miliary TB with spinal TB (Pott spine) and improvement of clinical features following treatment with antitubercular drugs.

## Case report

**Figure 1:**
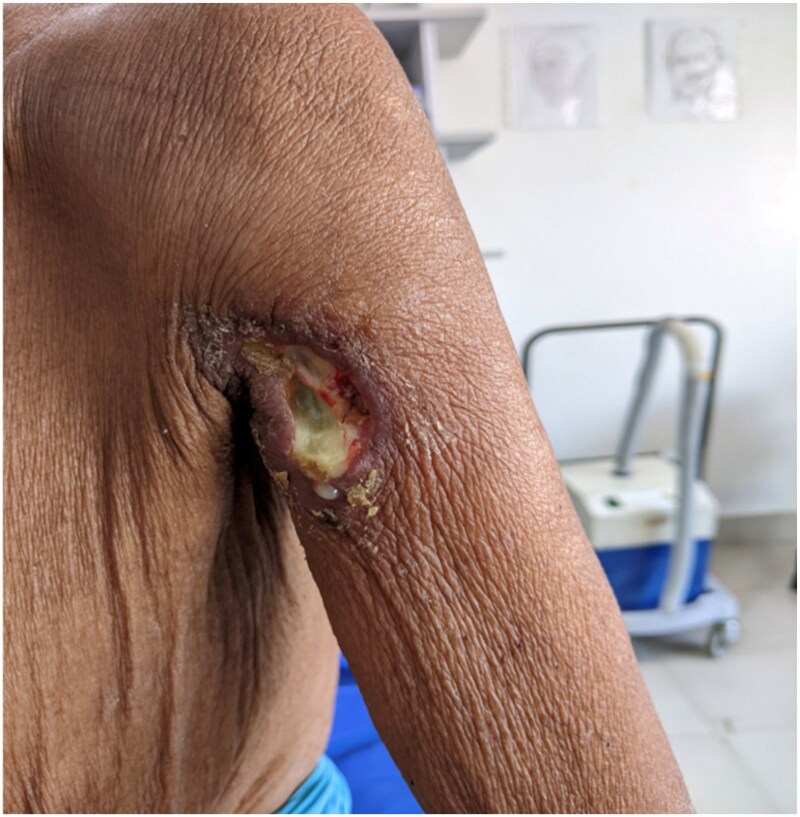
Nonhealing cutaneous ulcer with yellowish slough on the left upper arm.

The 67-year-old woman presented to the dermatology outpatient department with a 6-month history of multiple painful, nonhealing ulcers with yellowish discharge over the mons pubis and perivulvar area, along with a single ulcer on the left upper arm present for 5 months (Figure 1). The lesions began as firm, tender swellings that spontaneously ruptured to form ulcers.

On general examination, she appeared thin and poorly nourished, weighing approximately 38 kg with a body mass index of 17.5 kg m^−2^. Serum albumin was 2.8 g dL^−1^, consistent with protein-calorie undernutrition, a potential predisposing factor for disseminated TB. She denied any history of diabetes mellitus, chronic kidney disease or other chronic illnesses, and was not taking corticosteroids or other immunosuppressive medications. Serological tests for HIV1/2, hepatitis B, hepatitis C and syphilis were negative. The patient reported not having received BCG ­vaccination in childhood.

Cutaneous examination revealed 4–5 well-defined, painful ulcers with firm, indurated margins and yellowish discharge in the perivulvar region, each measuring approximately 1 × 2 cm. A solitary ulcer measuring 4 × 5 cm was present on the left upper arm, with irregular borders and a granulating base. No regional lymphadenopathy was detected.

Prior to presentation, the patient had been admitted to another department and received multiple courses of intravenous antibiotics without any clinical improvement.

## Results

**Figure 2:**
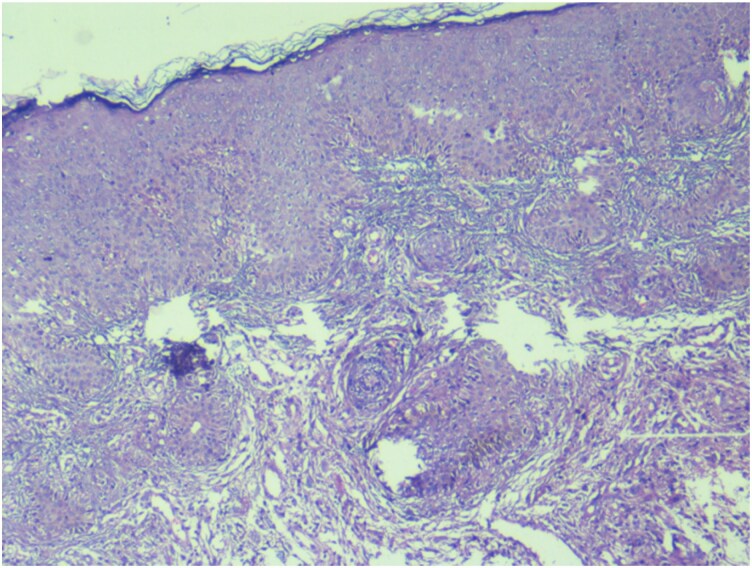
Histopathological image showing multiple foci of epithelioid-cell granulomas composed of aggregates of epithelioid histiocytes, lymphocytes and occasional plasma cells with few Langerhans-type multinucleated giant cells. Haematoxylin and eosin stain, original magnification ×40.

**Figure 3:**
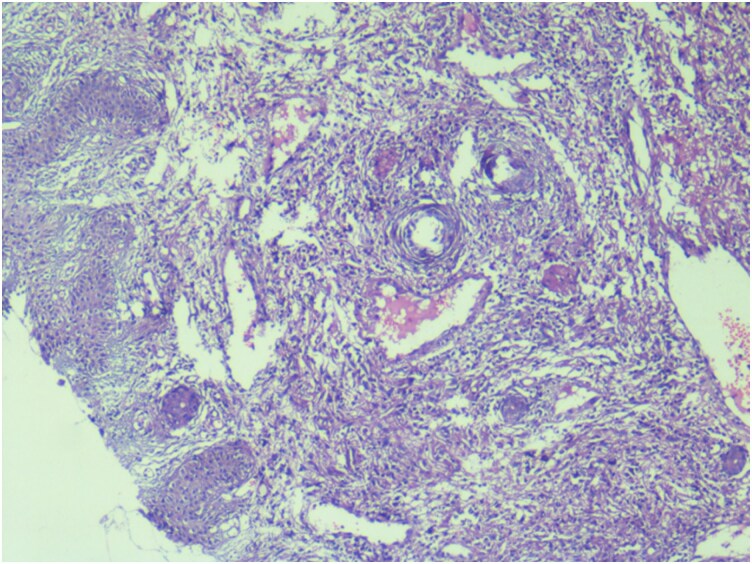
Multiple epithelioid cell granulomas comprising epithelioid histiocytes with surrounding lymphocytes and occasional plasma cells, along with few Langhans-type multinucleated giant cells (haematoxylin and eosin, ×40).

A punch biopsy taken from the edge of one ulcer revealed multiple foci of epithelioid-cell granulomas composed of aggregates of epithelioid histiocytes, lymphocytes and occasional plasma cells. Scattered Langhans-type multinucleated giant cells were also identified. Some granulomas exhibited central areas of amorphous eosinophilic necrosis consistent with caseation (Figures 2, 3). No fungal elements or atypical cells were observed. Ziehl–Neelsen staining for acid-fast bacilli was negative. However, tissue GeneXpert analysis from the ulcer on the left upper arm was positive for *M. tuberculosis*, and acid-fast bacilli were detected. Further drug resistance testing could not be performed due to unavailability of facilities.

Routine haematological and biochemical parameters were within normal limits, and the Mantoux test was negative. Sputum smear for acid-fast bacilli was noncontributory. No regional lymphadenopathy was observed.

**Figure 4:**
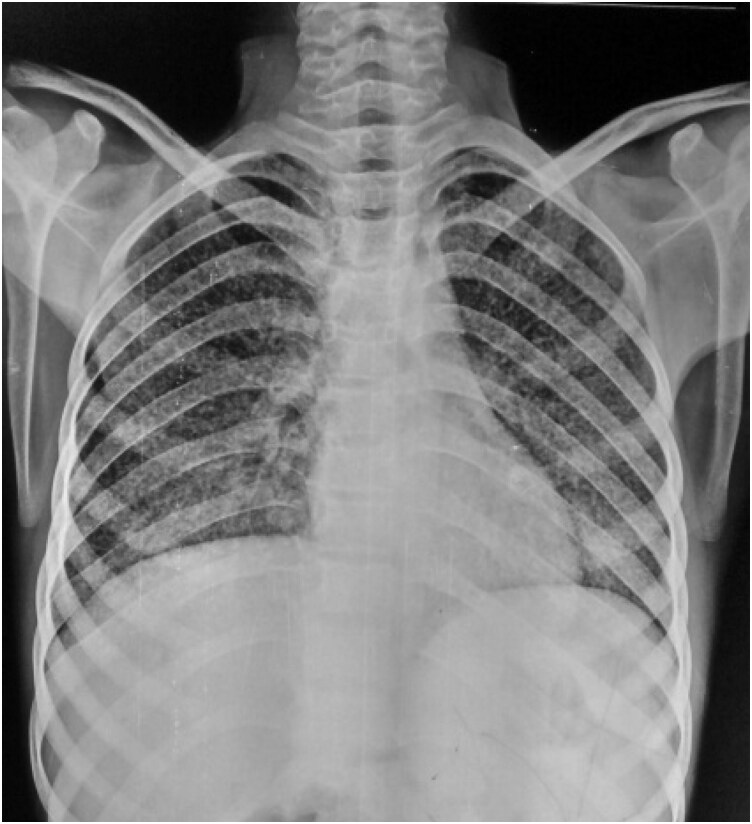
Chest radiograph (posteroanterior view) showing diffuse micronodular opacities throughout both lung fields.

Chest radiography demonstrated numerous bilateral pulmonary nodules suggestive of miliary TB (Figure 4). Given the patient’s report of persistent lower-back pain, spinal imaging was performed. X-ray and magnetic resonance imaging of the dorso­lumbar spine revealed findings consistent with spinal TB.

The patient was initiated on standard antitubercular therapy (ATT; isoniazid, rifampicin, ethambutol and pyrazinamide). After 1 month of treatment, there was notable clinical improvement, with significant reduction in ulcer size and resolution of discharge. Unfortunately, the patient was lost to follow-up beyond the first month, limiting the ability to assess complete resolution and the long-term outcome.

## Discussion

Cutaneous TB is a relatively uncommon manifestation of extrapulmonary TB, accounting for 1–1.5% of all cases of extrapulmonary TB.^1^ Among the various clinical types, miliary cutaneous TB is the least common, typically occurring secondary to haematogenous spread during systemic miliary TB.^8^ This form is associated with immunocompromised status, advanced age or chronic comorbidities, and is therefore considered a marker of disseminated disease.^7^

Clinically, miliary cutaneous TB can present with papules, nodules, pustules, plaques or ulcers that are often nonspecific and mimic other bacterial, fungal or malignant lesions.^9^ The ulcerative variant, as seen in our patient, may be mistaken for chronic bacterial or fungal infection, pyoderma gangrenosum or atypical mycobacterial infection. Because of such varied presentation, diagnosis is often delayed, leading to increased morbidity and dissemination to visceral organs such as lungs, liver, intestine or spine.^10^

Histopathological examination remains a cornerstone of diagnosis. The characteristic features include tuberculoid granulomas composed of epithelioid histiocytes, lymphocytes and Langerhans-type multinucleated giant cells, often with variable caseous necrosis.^5^ In our case, the presence of well-formed granulomas with focal caseation strongly suggested a tuberculous aetiology. Although Ziehl–Neelsen staining for acid-fast bacilli was negative, this finding is not uncommon, as cutaneous TB lesions may contain very few organisms. In such cases, a diagnosis relies on compatible clinical features, histopathology and response to ATT.

Sometimes the first sign of systemic miliary TB is cutaneous lesions.^10^ Similar situations where chronic, nonhealing ulcers were the presenting symptom prior to the diagnosis of widespread TB have been reported in a number of publications.^11^ The nonhealing lesion in our patient and the imaging results that followed, which showed miliary nodules in the lungs and spinal TB, highlighted the significance of cutaneous symptoms as indicators of systemic illness.

A combination of clinical, radiographic and microbiological assessment is frequently necessary for the diagnosis of miliary TB. Even though the tissue yield could be limited, confirmation can be aided by histopathology, staining for acid-fast bacilli, and molecular testing like GeneXpert or polymerase chain reaction.^12^ Our example highlights that when clinical and histological results strongly suggest TB, early empirical ATT can be warranted even in the absence of microbiological proof.

According to national and World Health Organization standards, the conventional treatment for miliary and cutaneous TB consists of an intense 6-month course of isoniazid, rifampicin, pyrazinamide and ethambutol, followed by continuing with isoniazid and rifampicin.^13,14^ Within 4 weeks of treatment, our patient showed ulcer healing and a significant reduction in systemic symptoms, confirming the diagnosis and treatment outcome. In cases where the spine is involved, early commencement of ATT is crucial to prevent additional spread and consequences, including vertebral damage or neurological issues.^15^ This instance emphasizes how difficult it can be to diagnose cutaneous miliary TB, especially in people who are immunocompetent who do not exhibit obvious pulmonary symptoms. Particularly in endemic regions, the occurrence of persistent, antibiotic-unresponsive ulcers should raise suspicions of atypical diseases, such as TB. Dermatologists are essential to identifying these atypical manifestations and enabling prompt referral for comprehensive assessment.

A limitation of this report is the absence of microbiological confirmation of *M. tuberculosis* through culture or molecular testing, primarily due to low bacillary load in cutaneous lesions and limited diagnostic resources. However, the combination of compatible clinical, histopathological and radiological findings, along with prompt therapeutic response, strongly supported the diagnosis.

In conclusion, cutaneous TB, particularly in its miliary form, remains a rare and often overlooked presentation of systemic TB.

## Data Availability

The data underlying this article are available in the article.
